# Engineered human B cells targeting tumor-associated antigens exhibit antigen presentation and antibody-mediated functions

**DOI:** 10.3389/fimmu.2025.1621222

**Published:** 2025-07-30

**Authors:** Alexander Boucher, Courtney Anderson, Rochelle Hinman, Molly Kindschuh, Jeremy Fung, Tiansu Wang, Isabella Klooster, Elise Kim, Caroline Roth, Michael Vander Oever, Bakhmala Khan, Natalie Zelikson, Yaron Vagima, Huseyin Saribasak, Lisa Santry, Leah Natasha Klapper, Shmuel Hess, Jill Mooney, Débora Rosa Bublik, Haley Laken, Adi Barzel, Philip Borden, Cherylene Plewa, Ana Maria Chadbourne, Devin Bridgen, Alessio D. Nahmad

**Affiliations:** ^1^ ElevateBio, Waltham, MA, United States; ^2^ Tabby Therapeutics, Watertown, MA, United States; ^3^ Life Edit Therapeutics, Durham, NC, United States; ^4^ Faculty of Medical & Health Sciences, Tel Aviv University, Tel Aviv, Israel; ^5^ Department of Biotechnology, Israel Institute for Biological Research, Ness Ziona, Israel; ^6^ Tabby Therapeutics, Ness Ziona, Israel; ^7^ Faculty of Life Sciences, Tel Aviv University, Tel Aviv, Israel; ^8^ The Samueli Integrative Cancer Pioneering Institute, Petah Tikva, Israel

**Keywords:** antibody, B cell, tertiary lymphoid structure (TLS), immune complex, antigen presentation, genome editing, cell engineering

## Abstract

B cell engineering represents a promising therapeutic strategy that recapitulates adaptive immune functions, such as memory retention, antibody secretion and affinity maturation in murine models of viral infection. These mechanisms may be equally beneficial in oncology. Recent studies have linked endogenous anti-tumor B cell immunity to favorable prognosis across multiple malignancies. Here, we present functional validation of human B cells engineered to target tumor-associated membrane and intracellular antigens. We demonstrate that engineered B cells express therapeutically relevant membrane B cell receptors that are secreted as antibodies upon differentiation. Additionally, engineered B cells take up tumor-associated antigens and demonstrate potent antigen presentation capabilities, while their secreted antibodies activate T cell responses via immune complexes and induce tumor-directed cytotoxic responses. B cell engineering to target tumor-associated antigens may thus have utility as a novel modality for solid tumor therapy.

## Introduction

Engineered adoptive immune cell therapies commonly harness immune effector cells such as T or NK cells for treating diverse pathologies (1–3). Chimeric Antigen Receptor (CAR) or engineered T Cell Receptor (TCR) therapeutic platforms are directed to either membrane-bound or intracellular targets, respectively. While highly specific and potent for treating rheumatologic diseases and hematological-malignancies (4–8), these engineered T cell therapies are susceptible to escape, exhaustion, and have diminished persistence when employed for solid tumor therapy (9–14). Some additional efficacy may be gained by activation of endogenous immune cells by coding for immune effectors such as cytokines. However, safety hurdles associated with triggering unspecific immune activity are concerning (15–17). In contrast, Tumor Infiltrating Lymphocyte (TIL) therapy leverages ongoing endogenous anti-tumor immune cells (18). TIL therapy is effective for solid tumors, but is associated with heavy manufacturing hurdles as highlighted by 35 years of clinical development (19–21). In addition, no effector immune cell is currently clinically approved for utilization without lymphodepletion prior to therapy. Lymphodepletion is believed to be required to enhance transplant rates and responses at the hefty price of ablating ongoing endogenous anti-tumor responses in already heavily treated patients. Therefore, an ideal immune cell platform therapy would allow recognition of target antigens regardless of subcellular location, capable of enhancing endogenous responses restricted to the tumor and be employable without lymphodepletion.

In this context, tumor-specific B cells emerge as a promising new modality. B cell infiltration leads to improved immune checkpoint blockade responses in melanoma and sarcoma (22–25). B cells may target multiple tumor-associated antigens and support T cell activity by means of antigen presentation (26, 27). By leveraging other immune cells, B cells improve *in situ* anti-tumor activity and form complex immune niches called Tertiary Lymphoid Structures (TLSs) (28–30). TLSs correlate with positive prognosis in several conditions (31–33). In TLSs, B cells undergo affinity maturation, to potentially counteract tumor escape (26, 34). Finally, recent B cell therapies for rare diseases were administered without lymphodepletion (35).

This evidence supports investigating the therapeutic potential of B cells engineered to express tumor-specific antibodies. Expression of the antibody as a B Cell Receptor (BCR) could allow the engineered B cells to infiltrate, proliferate and differentiate *in situ*, thereby supporting T cell reactions and TLS formation. Upon differentiation into plasmablasts or plasma cells, antibodies secreted by engineered B cells would further enable activation of anti-tumor responses by NK cells, macrophages, the complement system and dendritic cells.

Here, we evaluate the potential of B cells engineered to target intracellular or membrane-bound tumor antigens. We demonstrate that primary human engineered B cells acquire antigens for presentation to T cells, leading to their activation. Furthermore, engineered B cells produce soluble antibodies eliciting tumor cytotoxicity and facilitating antigen presentation. To our knowledge, this is the first report of engineered B cells targeting antigens from solid tumors demonstrating both antigen presentation as well as antibody-mediated cytotoxicity.

## Results

### Screening identifies guide RNAs that mediate high gene editing activity in human B cells and BCR expression following antibody gene knock-in

Antigen-specific B cells recognize antigens that are intracellular or membrane-bound. Upon activation, B cells elicit T cell responses through antigen presentation and trigger other immune cells activity via antibody-mediated functions (36, 37). An efficacious engineered B cell therapeutic would harness these capabilities and emulate expected downstream B cell activation processes, such as class switch recombination and somatic hypermutation. We and others previously achieved this by engineering the IgH locus (38–44) (**Supplementary Figure 1A**).

To optimize engineering rates at the J-C region of the IgH locus in human B cells, we aimed to use novel RNA-guided nucleases and we designed a panel of locus-specific guide RNAs (gRNAs) using a proprietary bioinformatics platform. Briefly, all potential protospacer adjacent motifs (PAMs) for a panel of proprietary RNA-guided nucleases, including Nuclease A, were identified in the intragenic region of the IgH locus, and the corresponding spacer sequences were extracted. These spacer sequences were then subjected to a preliminary off-target search to analyze whether the sites had a high degree of sequence homology to other regions of the human genome. gRNAs with excessive homology to other regions of the genome were dropped and not screened.

**Figure 1:**
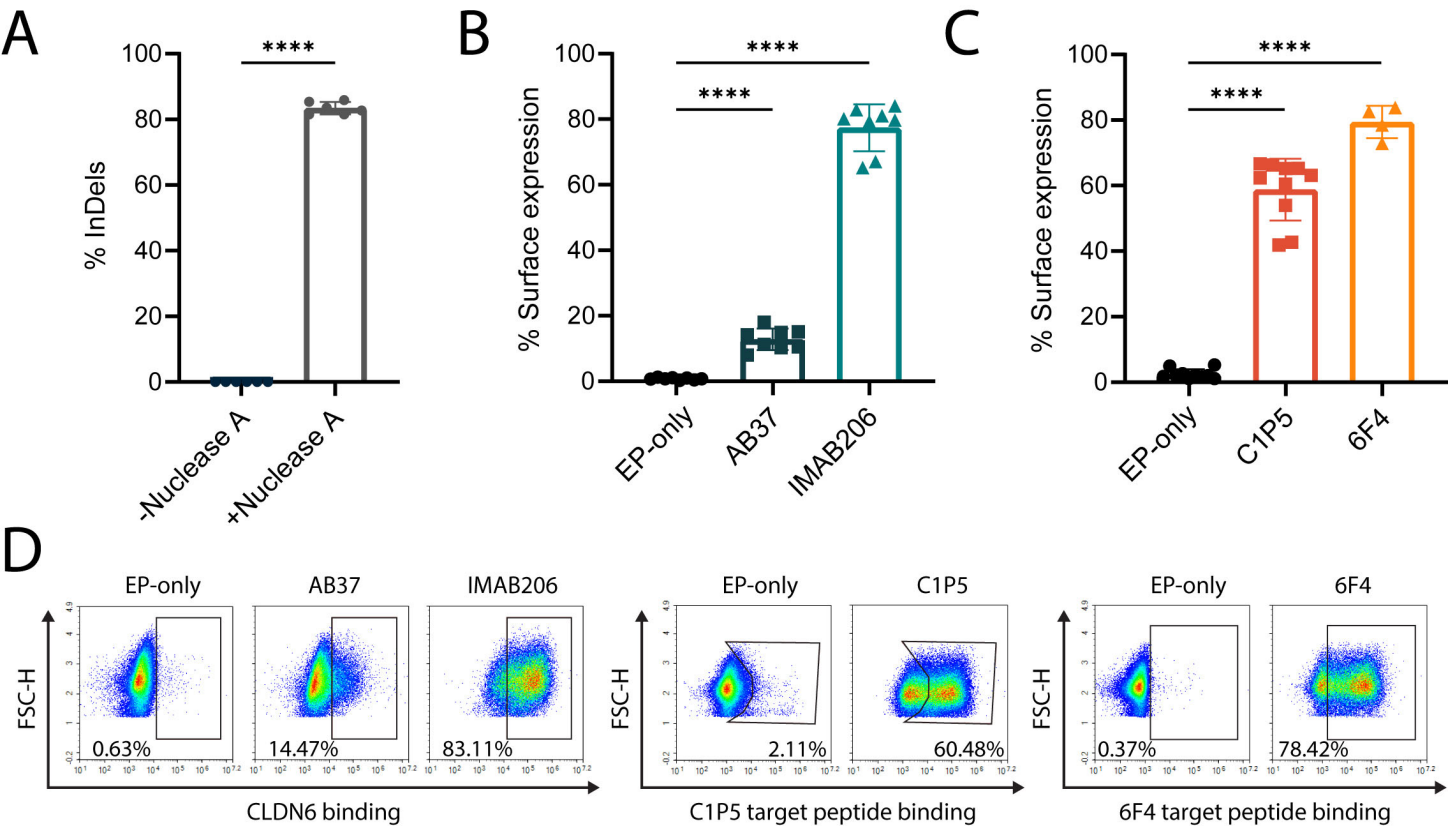
Engineering of primary human B cells with antigen-specific BCRs. **(A)** Percent of InDels in the targeted IgH locus of edited B cells with and without Nuclease A provided with Guide 10. ****=pv<0.0001, two-tailed unpaired t-test. **(B)** Percentage of surface expression of CLDN6-specific BCRs (AB37 and IMAB206), as measured by flow cytometry, compared to EP-only control B cells. Data are the mean of 6 experiments with 2 donors for IMAB206 and 4 experiments with 2 donors for AB37. **(C)** Same as **(B)** for E6-specific BCRs (C1P5 and 6F4). Data are the mean of 3 experiments with 3 donors for C1P5 and 2 experiments with 2 donors for 6F4. For **(A-C)** mean is indicated and error bars represent SD, each dot represents an independent experiment. For **(B, C)** ****=pv<0.0001 for one-way ANOVA with Tukey’s multiple comparison test. **(D)** Representative flow cytometry plots for **(B, C)** of surface BCR expression of engineered B cells.

Nominated gRNAs and their cognate nucleases were then screened for editing in primary human B cells and we identified several gRNAs for Nuclease A that consistently conferred greater than 70% on-target editing (insertions and deletions, InDels) at the desired locus (**Supplementary Figure 1B**). Therefore, Nuclease A was chosen as the lead RNA-guided nuclease for this target site. Of our five top gRNAs, we found that the spacer sequence of Guide 10 had significant overlap with the gRNA used in our previously successful work with Cas9 (38). We further validated Guide 10 in primary human B cells from the peripheral blood of several healthy donors and achieved up to 80% editing efficiency (**Figure 1A**). Therefore, we selected this guide for use with Nuclease A in subsequent experiments and designed corresponding donor cassettes.

### Human B cells are engineered at high efficiencies

We evaluated whether B cells engineered to express orthogonal antibodies could support recognition of cell surface or intracellular antigens. To narrow down therapeutically relevant target antigen candidates, we first selected malignancies where it had been demonstrated that the presence of B cells correlated with improved patient outcomes (31, 45–47). We then focused on a list of targets that were present in diseases with high unmet medical need and selected tumor-specific antigens such as foreign or oncofetal antigens. For the intracellular target, we selected E6, a protein from human papilloma virus (HPV). E6 is a tumor-specific antigen directly associated with transformation of infected cells. HPV is known to be associated with head and neck cancers in which B cell activity has been correlated with positive prognosis (48). For the membrane-bound antigen, we selected CLDN6, an oncofetal antigen. Therapeutics targeting CLDN6 have demonstrated initial clinical efficacy given its low expression in healthy tissues and tumor-restricted expression (49, 50).

We then designed Adeno-Associated Vector (AAV) constructs for the expression of two antibody sequences specific for either E6 (C1P5 and 6F4) or CLDN6 (IMAB206 and AB37). The antibodies, as previously characterized, target separate epitopes on each antigen (51–54). We packaged the corresponding constructs in recombinant AAV serotype 6 and, together with Nuclease A and Guide 10 delivered by electroporation, engineered human B cells from the peripheral blood of healthy donors (38, 42). For each of the targeted antigens, we reached ~75% on-target engineering efficiency as quantified by flow cytometry for the detection of surface antigen specific BCR (**Figures 1B–D**).

### Engineered human B cells express functional BCRs and secrete antibodies targeting membrane-bound or intracellular tumor antigens

**Figure 2:**
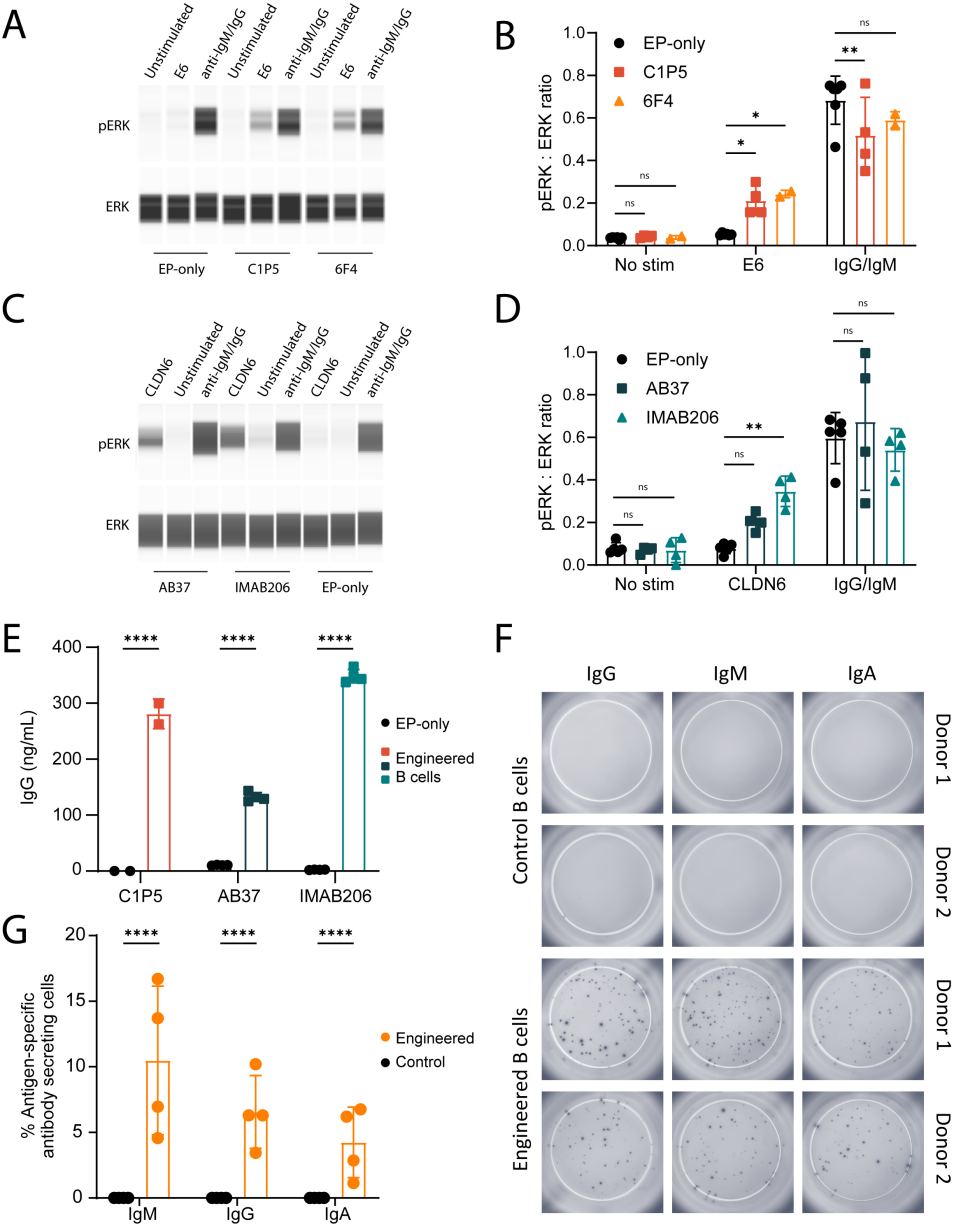
Engineered B cells activate BCR signaling cascades and secrete isotype-switched antigen-specific antibodies. **(A)** Representative immunoblot of B cells engineered to express either C1P5 or 6F4 E6-binding BCRs, stimulated with recombinant E6 protein, anti-IgM/IgG protein control or unstimulated. Protein extracts were analyzed using anti-pERK antibody (top panel) or anti-total ERK antibody (bottom panel). **(B)** Quantification of mean ratio expression of ERK to pERK as in **(A)** ns=pv>0.05, *=pv<0.05, **=pv<0.01 for two-way ANOVA with Dunnett’s multiple comparisons test. **(C)** Same as A for the CLDN6-binding BCRs AB37 or IMAB206. Cell stimulation occurred with recombinant CLDN6 protein. **(D)** Quantification of experiments as in **(C)** For **(A-D)**, data representative of 2–3 experiments with 2 donor cells. ns=pv>0.05, **=pv<0.01 for two-way ANOVA with Dunnett’s multiple comparisons test. **(E)** Quantification of IgG antibodies in the supernatants of engineered B cells expressing either C1P5, AB37 or IMAB206, by ELISA. ****=pv<0.0001 for two-way ANOVA with Šídák’s multiple comparisons test. **(F)** Representative ELISPOT images of E6-specific IgM, IgG and IgA secreted from B cells engineered to express the anti-E6 6F4 antibody. **(G)** Quantification of data as in **(F)** as normalized to the total amount of cells seeded in the wells. ****=pv<0.0001 for two-way ANOVA with Šídák’s multiple comparisons test. For **(E-G)** pooled data from 2–3 experiments with 2–4 donor cells. For **(B, D, E)**, **(G)** mean and error bars are indicated, each dot represents an independent experiment.

Upon antigen binding, a functional BCR will activate a signaling cascade which results in phosphorylation of ERK (55, 56). To evaluate this in engineered B cells, we stimulated the cells with their corresponding antigens, and lysates were analyzed by immunoblot using antibodies specific for phosphorylated ERK. Engineered cells stimulated with the relevant antigen led to phosphorylation of ERK but not control B cells nor non-stimulated cells (**Figures 2A–D**).

We further evaluated whether our engineered B cells secreted antigen-specific antibodies of multiple isotypes. Antigen-specific ELISA and ELISPOT assays were designed to evaluate secretion of IgG, IgA and IgM. Our data show that engineered B cells with E6- and CLDN6-specific antibodies secrete antigen-specific IgGs (**Figure 2E**). The numbers of IgM, IgG and IgA antibody-secreting cells were similar between engineered and non-engineered B cells, demonstrating that the engineering process does not affect differentiation or isotype expression (**Supplementary Figures 1C, D**). Additionally, B cells engineered to express the 6F4 antibody produced E6-specific IgG, IgM and IgA (**Figures 2F, G**). Thus, engineered primary human B cells secrete poly-isotypic antibodies upon differentiation into antibody secreting cells.

### Antibodies secreted by engineered B cells elicit antibody-mediated functions when targeting membrane-bound tumor-associated antigens

**Figure 3:**
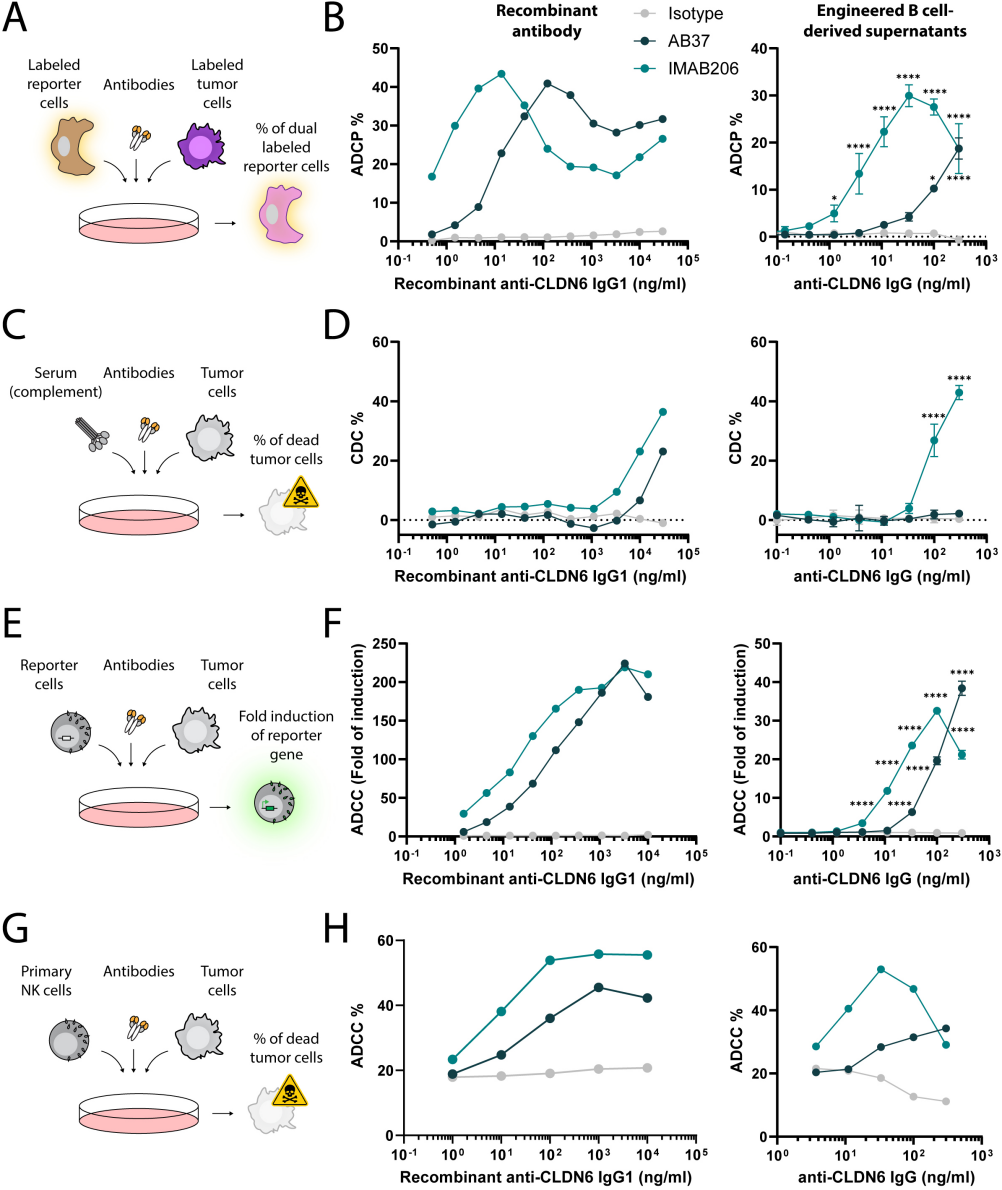
Antigen-specific IgGs secreted by engineered B cells trigger Fc-mediated functions. **(A)** Schema depicting the flow cytometry-based ADCP assay. Labeled reporter cells were incubated with labeled tumor cells and either recombinant antibodies or concentrated antibodies from the supernatant of engineered B cells. The frequency of dual labeled reporter cells was analyzed. **(B)** ADCP response of recombinant IgG1 antibodies (left) and concentrated engineered B cell supernatants (right) for Isotype, AB37, and IMAB206 against CLDN-6-expressing PA-1 cells determined by flow cytometry. *=pv<0.05, ****=pv<0.0001 for two-way ANOVA with Dunnett’s multiple comparisons test to the isotype control. **(C)** Schema depicting the flow cytometry-based CDC assay. Tumor cells were incubated with sera and either recombinant antibodies or concentrated antibodies from the supernatant of engineered B cells. The frequency of dead tumor cells was analyzed. **(D)** CDC response of recombinant IgG1 antibodies (left) and concentrated engineered B cell supernatants (right) for Isotype, AB37, and IMAB206 against CLDN-6-expressing PA-1 cells, as determined by flow cytometry. ****=pv<0.0001 for two-way ANOVA with Dunnett’s multiple comparisons test to the isotype control. **(E)** Schema depicting the ADCC reporter assay. Reporter cells were incubated with tumor cells and either recombinant antibodies or concentrated antibodies from the supernatant of engineered B cells. The fold induction of the luciferase reporter gene in the reporter cells was analyzed. **(F)** ADCC response of recombinant IgG1 antibodies (left) and concentrated engineered B cell supernatants (right) for Isotype, AB37, and IMAB206 against CLDN-6-expressing PA-1 cells determined by luciferase fold of induction. ****=pv<0.0001 for two-way ANOVA with Dunnett’s multiple comparisons test to the isotype control. **(G)** Schema depicting the flow cytometry-based ADCC assay. Primary NK cells were incubated with tumor cells and either recombinant antibodies or concentrated antibodies from the supernatant of engineered B cells. The frequency of dead tumor cells was analyzed. **(H)** ADCC response of recombinant IgG1 antibodies (left) and concentrated engineered B cell supernatants (right) for Isotype, AB37, and IMAB206 against CLDN-6-expressing PA-1 cells, as determined by flow cytometry. For the engineered B cell-derived supernatants, data are representative of technical replicates and two **(B)** or one **(D-F)** experiments using two independent donors to generate supernatants or one experiment using one donor to generate supernatants **(H)**. Error bars indicate SD.

Membrane-bound antigens are expected to enable antibody-mediated cytotoxic functions. Therefore, next, we determined whether the secreted antibodies produced by engineered B cells targeting the membrane-bound antigen CLDN6 had the capacity for Fc-mediated functions, such as antibody-dependent phagocytosis (ADCP), complement-dependent cytotoxicity (CDC), and antibody-dependent cell cytotoxicity (ADCC). Recombinant IgG1 control antibodies and concentrated supernatants from engineered B cell culture were tested against the CLDN6-expressing tumor cell line PA-1, which express CLDN6 at high levels (**Supplementary Figures 1E, F**). Engineered B cell derived antibodies elicited ADCP (**Figures 3A, B**), CDC (**Figures 3C, D**), and ADCC (**Figures 3E–H**) responses as indicated by both reporter assays and cytotoxicity of target cells. At high antibody concentrations, the hook effect, as also observed by others, led to slightly decreased ADCP, ADCC and CDC efficiency (57–59). Overall, these results demonstrate that engineered B cells targeting membrane-bound tumor antigens secrete functional IgG antibodies.

### B cells engineered to target intracellular antigens act as antigen-presenting cells to activate class II-restricted T cells

A differentiating factor for B cells as therapeutics, compared to monoclonal antibodies, would be the ability to induce T cell activation via antigen presentation. It has previously been shown that B cells that have antigen presenting capabilities are characterized by low expression of CD21 and high expression of CD86 (36). We evaluated the phenotype of our engineered B cells and found that after seven days in culture, most cells had differentiated to switched memory (IgD^-^CD27^+^), with about 30-40% of cells bearing a plasmablast phenotype (CD27^+^CD38^+^). Interestingly, almost all engineered B cells carried the antigen-presenting phenotype reported, as most cells were CD21^-^CD86^+^ (antigen presenting cells, APC) at the end of the culture process (**Supplementary Figure 2A**).

**Figure 4:**
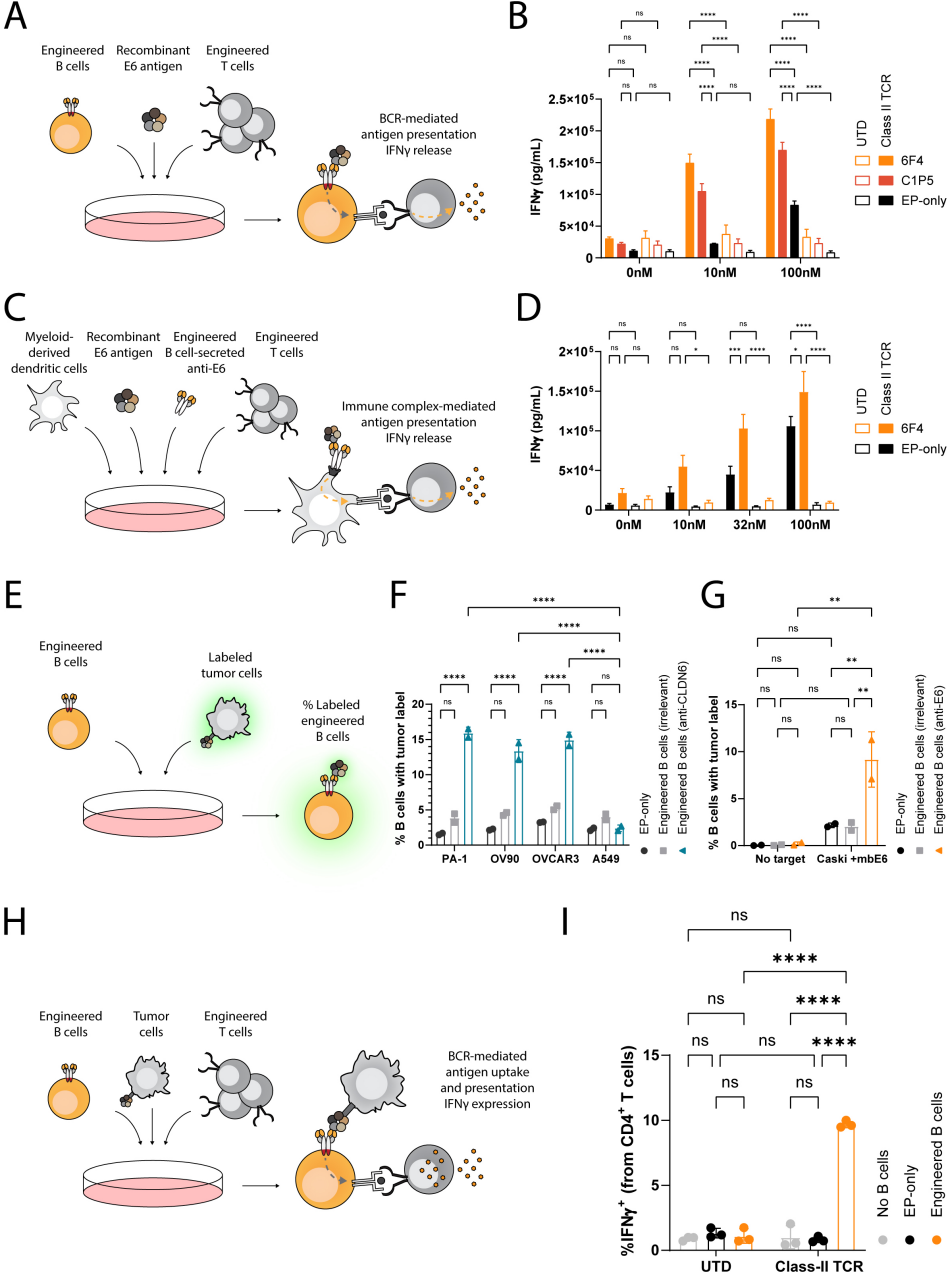
Engineered B cells and immune complexes taken up by dendritic cells present E6 antigen to activate E6-specific MHC class II-restricted T cells. **(A)** Schema depicting the engineered B cell antigen presentation assay. B cells, either non-engineered (EP-only) or engineered to express anti-E6 antibodies (6F4 or C1P5), are loaded with recombinant E6 antigen at multiple concentrations and then incubated with T cells, either engineered (Class II TCR) or non-engineered (UTD) to express an anti-E6 TCR. The concentration of IFNγ in the supernatants is then analyzed by ELISA. **(B)** ELISA for IFNγ as in **(A)** Data representative of 2 experiments with 2 donors. Bars and error bars represent mean and SD. ns=pv>0.05, ****=pv<0.0001 for two-way ANOVA with Šídák’s multiple comparisons test. **(C)** Schema depicting the immune complex-mediated antigen presentation assay. Immune complexes were formed with concentrated antibodies from the supernatants of B cells, either non-engineered or engineered to express anti-E6 antibodies, and recombinant E6 antigen at multiple concentrations. These were then incubated with myeloid-derived dendritic cells and then co-cultured with T cells, either engineered or non-engineered to express an anti-E6 TCR. The concentration of IFNγ in the supernatants is then analyzed by ELISA. **(D)** ELISA for IFNγ as in **(C)** Data representative of 2 experiments with 2 donors. Bars and error bars represent mean and SD. ns=pv>0.05, *=pv<0.05, ***=pv<0.001, ****=pv<0.0001 for two-way ANOVA with Šídák’s multiple comparisons test. **(E)** Schema depicting the flow cytometry-based engineered B cell membrane-bound antigen uptake assay. Engineered or non-engineered B cells are incubated with labeled tumor cells either expressing or not expressing the targeted antigen. The frequency of B cells with the tumor label is then analyzed by flow cytometry. **(F)** Flow cytometry results as in **(E)** for B cells engineered to express anti-CLDN6 IMAB206 (blue) or anti-E6 (6F4) antibodies (grey). ns=pv>0.05, ****=pv<0.0001 for two-way ANOVA with Šídák’s multiple comparisons test. **(G)** Flow cytometry results as in **(E)** for B cells engineered to express anti-E6 6F4 antibodies (orange), or anti-CLDN6 IMAB206 antibodies (grey). ns=pv>0.05, **=pv<0.01, for two-way ANOVA with Tukey’s multiple comparisons test. **(H)** Schema depicting a flow cytometry-based engineered B cell antigen presentation assay. B cells, either non-engineered or engineered to express anti-E6 antibodies, were incubated with tumor cells, engineered to express the membrane-bound E6 antigen, and with T cells, either engineered or non-engineered to express an anti-E6 TCR. The frequency of T cells expressing intracellular IFNγ was then analyzed by flow cytometry. **(I)** Flow cytometry results as in **(H)** for B cells engineered to express anti-E6 6F4 (orange) antibodies. Data representative of 3 experiments using one donor. Bars and error bars represent mean and SD. ns=pv>0.05, ****=pv<0.0001, for two-way ANOVA with Tukey’s multiple comparisons test.

APCs internalize antigens for proteolysis to allow derived peptides to be presented on Major Histocompatibility Complexes (MHC). T cells recognize these specific peptide/MHC complexes via their TCRs. To demonstrate APC capabilities of engineered B cells, we employed E6-specific engineered B cells and investigated antigen presentation to autologous, engineered, E6-specific T cells. Specifically, we engineered primary human T cells to express a class II-restricted E6-specific TCR. This TCR recognizes the HPV16-E6_1–15_ peptide in the context of the MHC Human Leukocyte Antigen (HLA) DQA1*01:02/DQB1*05:02 (60) (**Supplementary Figure 2B**). In parallel, we isolated B cells from the same donors and engineered them to express either the anti-E6 C1P5 or 6F4 antibodies (**Supplementary Figure 2C**). We used B cells that were electroporated in the absence of AAV6 (EP-only) as controls. Engineered or control B cells were protein-loaded by incubating with the soluble E6 protein at a concentration of 0, 10 or 100nM. Finally, we co-cultured the engineered T cells with the E6-loaded engineered B cells and analyzed secretion of IFNγ (**Figure 4A**). Importantly, E6-specific engineered B cells elicited potent T cell responses via antigen presentation, as indicated in co-cultures with engineered T cells (**Figure 4B**). Specifically, E6 at a concentration of 10nM enabled engineered B cells, but not non-engineered B cells, to trigger IFNγ secretion to concentrations reaching above 100ng/ml with both BCRs. At 100nM, while engineered B cells elicited much higher IFNγ secretion than non-engineered B cells, some IFNγ secretion could be detected in EP-only co-cultures, indicative of BCR-independent antigen uptake at high concentrations. In co-cultures with untransduced (UTD) T cells, minimal IFNγ secretion was observed with any B cells or antigen concentration (**Figure 4B**). EP-only control B cells could activate TCR engineered T cells when provided with peptide pools instead of the full E6 antigen and intracellular staining for IFNγ of transduced CD4 T cells corroborated ELISA concentrations (**Supplementary Figures 3A–C**). Together, these results highlight that engineered B cells utilize their BCR to facilitate antigen presentation leading to CD4 T cell activation.

### Engineered B cells secrete antigen-specific antibodies that activate T cells via immune complexes

Secreted antibodies may bind soluble antigens. These immune complexes are then acquired by Fc receptor expressing APCs, including dendritic cells. Subsequently, the APCs internalize the immune complexes to facilitate presentation of the captured antigen to T cells, enabling an additional mechanism driving anti-tumor immunity (61). We explored whether dendritic cells could take up E6-IgG immune complexes and present them to class II-restricted anti-E6 T cells. For these experiments, we prepared primary human myeloid-derived dendritic cells and preloaded them with immune complexes that had been formed with supernatants from engineered B cells targeting E6, or supernatant of control EP-only B cells, and increasing concentrations of E6 (**Supplementary Figure 3D**). We then co-cultured these dendritic cells with UTD or class II-restricted anti-E6 T cells (**Figure 4C**). Engineered B cells expressing the 6F4 binder were identified as robust anti-E6 candidates and were therefore selected. Unsurprisingly, UTD T cells did not secrete significant amounts of IFNγ in any of the conditions tested and anti-E6 T cells secreted IFNγ when dendritic cells were incubated with E6 protein, but not when incubated without E6. Importantly, IFNγ levels were much higher with E6 immune complexes formed with supernatants from engineered B cells, but not with supernatants from EP-only B cells (**Figure 4D**). Therefore, immune complexes formed between antibodies secreted by engineered B cells and tumor antigen facilitate antigen-specific T cell activation when taken up by dendritic cells.

### B cells engineered to target membrane tumor antigens activate class II-restricted T cells

Beyond soluble antigens, B cells can perform BCR-mediated trogocytosis following binding to membrane-bound antigens (62). To establish whether engineered human B cells can take up antigen from the membrane of tumor cells, we labeled the membranes of CLDN6-expressing tumor cell lines (PA-1, OVCAR3, and OV90) and a CLDN6-negative tumor line (A549) with a membrane intercalating dye. We then compared engineered B cells expressing a CLDN6-specific BCR to engineered B cells expressing an irrelevant BCR for their ability to take up the label (**Figure 4E**). Label uptake was detected only in the combination of B cells engineered to express CLDN6-specific BCRs and tumor cell lines expressing CLDN6 (**Figure 4F**). Concordantly, E6-specific engineered B cells, but not EP-only controls, were also able to take up membrane of labeled Caski cells engineered to express E6 tethered to the cell surface (Caski + mbE6) (**Figure 4G**).

To demonstrate that membrane-bound antigen uptake also leads to subsequent antigen presentation, we co-cultured antigen-specific B and T cells in the presence of Caski + mbE6 tumor cells and performed intracellular IFNγ staining (**Figure 4H**). E6 TCR T cells produced IFNγ when cultured in the presence of E6-specific engineered B cells but not when UTD T cells or EP-only B cells were present (**Figure 4I**). To our knowledge, this is the first demonstration of engineered B cells acquiring tumor-associated antigens directly from the membrane of tumor cells for presentation to T cells. The acquisition of tumor antigen directly from tumor cells by engineered B cells leading to the potentiation of T cell activity highlights that engineered B cells could be employed for genesis of TLS.

## Discussion

Improving *in situ* immune responses to tumors is a challenge of modern medicine. Immune checkpoint inhibitors spearheaded this approach and demonstrated high rates of responses in multiple tumor types, particularly in the context of patients with ongoing immune responses (63). Beyond the state-of-the-art, novel strategies need to be employed to improve clinical responses. B cells are multi-faceted immune cells (45). Indeed, B cells infiltrate tumors (33), recruit other immune cells to form TLS (26), improve affinity towards tumor-associated antigens over time (34), and perform systemic monitoring over the course of a lifetime (64). As the backbone of TLS, B cells may be harnessed and engineered to gain tumor antigen specificity for therapeutic capability.

Here, we showcased human engineered B cells and their multiple effector modalities. We demonstrated that B cells may be engineered to express antibodies targeting therapeutically relevant intracellular or membrane-bound antigens. Antibodies targeting membrane-bound antigens are secreted by differentiated engineered B cells and elicit tumor cell cytotoxicity via ADCP, CDC and ADCC. Furthermore, engineered B cells take up soluble and membrane-bound tumor-associated antigen for presentation and activation of T cells. Antigen-specific T cell activation is additionally achieved through immune complex formation, which enhances antigen uptake by dendritic cells. Importantly, membrane-bound antigens from tumor cells are internalized by B cells for potentiation of CD4 T cells. Of note, we utilized a limited amount of donor samples due to the requirement of a specific haplotype matching the TCR employed. Additional studies may therefore aim at characterizing additional haplotype and TCR pairs in the context of B cell-mediated, antigen-specific presentation. In this effect, an independent study reveals that mouse engineered B cells may enable polyclonal T cell activation, including CD8 T cell activation (65). Beyond therapy, B cell engineering may be employed to characterize and compare antibody-mediated functions in the context of B cell expression. Overall, our results highlight that engineered B cells may be harnessed as a novel modality for cancer therapy to potentially elicit broad immune responses via multiple pathways.

Comprehensive characterization of tumor antigens is required to define an ideal engineered B cell target. Membrane-bound and intracellular tumor-associated antigens may be downregulated, potentially reducing activity. This is particularly of relevance to E6, which was demonstrated to have little to no expression in HPV-induced human cancers (66). For membrane-bound antigens, shedding may reduce and exhaust effector functions (67) and T cell engager molecules may offer a more direct means of inducing T cell based responses on tumors (68). From a safety perspective, additional characterization of RNA-guided nucleases needs to be performed to avoid genotoxicity (69, 70). Furthermore, B cell malignancies following IgH translocations may occur (71), thus gene edited B cells needs to be carefully monitored in the clinic, or should aim at employing nuclease-free alternatives (72, 73). Multiple groups developed strategies to overcome mispairing of the transgenic antibody with the endogenous chains (38–40, 74, 75), still the effect of these events in relation to off-targeting is not well understood. Finally, deeper characterization of engineered B cell phenotypes in cell cultures (76), and of possible changes occurring following adoptive cell transfer (77), needs to be performed to avoid unwarranted B cell activity (78) and to improve B cell derived therapeutic outcomes (79). In this study, we focused on *in-vitro*, pre-clinical characterization of engineered B cells. Due to reliance on other immune effector cells for optimal activity and limited TLS formation in mice models recipient of human immune and tumor cells, concrete therapeutic benefit of engineered B cells may be best assessed in larger animal models (80) and in the presence of a cancer vaccine to stimulate T cells and B cells at the same time (49).

The potential of administering engineered B cells in the absence of lymphodepletion, as has been demonstrated for a related B cell therapy (35), has significant implications for safety and burden of care. In addition, B cell therapies may be conveniently suppressed by conventional therapies, if unwarranted effects are detected in the clinic (81). Finally, *in vivo* B cell engineering may support therapies for cancer and beyond in a scalable manner (42). Engineered B cell therapeutics thus emerge as a novel modality to address unmet medical needs for treating solid tumors.

## Materials and methods

### Reagents and cell lines

Antibodies for flow cytometry, intracellular staining, and anti-IgG/IgM were from BioLegend. Nuclease A and sgRNA were from IDT. E6 Peptivator was from Miltenyi. Recombinant E6 protein was from R&D Systems. B cell isolation kit was from StemCell Technologies or Miltenyi. Reagents for detection of secreted IFNγ were from Mesoscale Discovery (MSD). CLDN6 VLP, GFP CLDN6 VLP and isotype control VLP were from ACRObiosystems. Anti-phospho-ERK (Phospho-p44/42 MAPK Erk1/2) and anti-ERK antibody (p44/42 MAPK Erk1/2 were from Cell Signaling Technology. Anti-rabbit detection and fluorescent separation modules for Jess was from Protein Simple. Cell lines A549 (CRM-CCL-185), Caski (CRM-CRL-1550), OV90 (CRL-3585), OVCAR3 (HTB-161), PA-1 (CRL-1572), and THP-1 (TIB-202) cells were purchased from ATCC.

### Guide identification and screening

Guides were designed using Life Edit’s proprietary computational pipeline and then synthesized (IDT). Guides were screened in primary human B cells (STEMCELL Technologies) that were cultured in ImmunoCult-XF™ B cell base medium (STEMCELL Technologies) containing 1% penicillin-streptomycin (Gibco) and stimulated with ImmunoCult ACF human B cell expansion supplement (STEMCELL Technologies), following manufacturers guidelines. Cells were cultured at 37°C and 5% CO_2_ for 72 hours before electroporation to allow the cells to activate and recover from freeze thawing.

After 3 days, B cells were nucleofected with Nuclease A mRNA (TriLink) and gRNAs using an Amaxa 4D X-unit (Lonza). Nucleofections were performed in P3 buffer following manufacturer recommendations in a 20µL cuvette, using 2µg of mRNA, 4µg of gRNA, and 5x10^5^ cells per nucleofection. Program EO-117 was used. After nucleofection the cells were cultured for 4 days at 37°C and 5% CO_2_ before genomic DNA was harvested for DNA analysis using a NucleoSpin96 tissue kit (Machery Nagel).

Genomic DNA was subjected to next-generation sequencing following standard Illumina protocols. Amplicon sequencing was performed using target specific PCR1 primers designed to flank the region of interest, including Nextera adapters (**Supplementary Table 1**) using Phusion High-Fidelity DNA polymerase (ThermoFisher Scientific), and the following program: 98°C., 1 min; 30 cycles of [98°C., 10 sec; 62°C., 15 sec; 72°C., 30 sec]; 72°C., 5 min; 12°C., hold. PCR 2 was performed with primers containing Illumina Nextera Read 1 and Read 2 transposase adapter overhangs, using the following program: 98°C., 1 min; 35 cycles of [98°C., 10 sec; 67°C., 15 sec; 72°C., 30 sec]; 72°C., 5 min; 12°C., hold. Libraries were prepared following Illumina protocols and sequenced on an Illumina MiSeq targeting 100,000 on-target reads.

NGS data were analyzed using a modified CRISPResso pipeline (82), with custom parameters. Sequencing reads underwent a rigorous quality control analysis that includes minimum read count and alignment thresholds. Only reads passing this analysis were used to determine the percent InDels at the on-target site.

### Construct design

Constructs were designed as previously demonstrated (38, 42). In short, donor cassettes coding for an antibody express a full light chain and the variable fragment of the heavy chain. The variable domains are preceded by IgK and IgH signal peptides. The bi-cistronic cassette is separated by a 2A peptide and is spliced with the endogenous constant segments upon on-target integration utilizing the intronic side of a splice donor. The coding sequences were manually optimized to improve expression in human cells, incorporate somatic hypermutation hotspots, remove cryptic splice sites and enhance ssAAV production. Donor cassette sequences are available on request. Recombinant AAV-6 were produced by ElevateBio or PackGene.

### Amplicon Seq

Cell samples that were electroporated (but where AAV was not added) were pelleted and DNA extracted. Primers were designed to PCR-amplify the region where nuclease activity is expected, plus several hundred flanking bases. Following successful enrichment of the target region, a second round of PCR is used to attach Illumina sequencing adapters and sample specific barcodes. Samples were pooled in equivalent ratios and sequenced using an Illumina MiSeq or NextSeq in the 2x250 or 2x300 configuration. Reads were demultiplexed using sample specific barcodes and trimmed to remove poor quality sequences. Trimmed reads were aligned to the target amplicon sequence and QC metrics including the number of reads aligned and the percent of reads aligned were assessed. For each sample, reads sharing identical sequences are collapsed, marked with an outcome(s) (wildtype, insertion, deletion, or substitution), and summarized to obtain estimates of percent editing within the sample, which is represented as % InDel.

### B cell engineering

Leukopacks carrying healthy donor apheresis were washed and labeled with CD19 microbeads (Miltenyi). Labelled cells were separated into CD19 B cells and unlabeled T cells on a CliniMACS Plus system. Resulting cells were cryopreserved in 50% CS10 diluted in plasmalyte A. B cells were seeded in Immunocult human B cell expansion kit culture medium (Stem Cell Technologies). ACF human B cell expansion supplement was added to B cell Base medium at 2% final concentration (complete Immunocult media) and cells were resuspended at 0.25e6 cells/mL and incubated at 37°C and 5% CO_2_ for 3 days. After 3 days, cells were electroporated with the NEON transfection system (Thermo Fisher Scientific). Cells were electroplated at a concentration of 5e5 cells/10 uL using 10 or 100 uL NEON tips with NEON Buffer R; 2 μg sgRNA and 1 μg Nuclease A mRNA was used per 5e5 cells electroporated. Cells were electroporated with the following NEON settings: 1500 V, 20 MS, 1 pulse. Electroporated cells were then replated at 5e5 cells/1 mL of complete Immunocult media, followed by the addition of AAV. The MOI was determined for different AAVs by a titration to optimize for expression and cell health. Cells were incubated for 24 hours and then fed with an additional 1 mL of complete Immunocult media and incubated for an additional 3 days before surface expression was assessed by flow cytometry on day 7.

### Detection of BCR by flow cytometry

B cells engineered with C1P5 and 6F4 BCRs were detected using biotinylated peptides corresponding to the C1P5 E6 epitope (LKFYSKISEYRHYCYSLYGT) and 6F4 E6 epitope (MHQKRTAMFQDPQERPRKLP), followed by fluorescently labeled streptavidin. As negative controls, we used mutated versions of the peptides. All peptides were synthesized at Genscript. For CLDN6 BCR detection, we used commercially available CLND6 and isotype VLPs conjugated with GFP (ACROBiosystems). Cell staining was performed by first incubating cells in live/dead Zombie Violet dye (BioLegend), Fc blocked with Trustain FcX (BioLegend), followed by biotinylated peptide or VLPs in FACS staining buffer (Invitrogen). For peptide staining, cells were washed, incubated with conjugated Streptavidin-PE (Miltenyi) and washed again prior to acquisition.

### ERK phosphorylation

B cells that were engineered with C1P5, 6F4, AB37, or IMAB206 were stimulated with recombinant E6 protein (for C1P5 or 6F4 engineered B cells) or CLDN6 VLP (for AB37 and IMAB206 engineered B cells), anti-IgM/IgG protein or left unstimulated. Cells were lysed and protein extracts analyzed by immunoblot using anti-phospho-ERK (Phospho-p44/42 MAPK Erk1/2) antibody or anti-total ERK (p44/42 MAPK Erk1/2) antibody (Cell Signaling Technology). Cell lysate concentrations were measured using the Pierce BCA Protein Assay Kit (Thermo Fisher Scientific). Western blotting was carried out using anti-rabbit detection and fluorescent separation and the Jess system (Protein Simple). Levels of phosphorylated ERK were determined by calculating the ratio of the intensity of phosphorylated and total ERK bands, as measured by densitometry.

### E6 TCR-T engineering

Constructs carrying the variable α and β chains of a MHC Class II-restricted E6-specific TCR (60) and murine TCRα and TCRβ constant chains were designed and cloned in a transfer plasmid. Lentiviral particles were generated by transducing HOS cells with transfer, packaging, and envelope plasmids, and titer was measured. PBMCs from donors carrying the MHC Class II haplotype of DQA1*01:02/DQB1*05:02 were transduced with lentiviral particles containing the E6-specific construct (Class II TCR) or left untransduced and expanded through activation of CD3 and CD28. Engineered T cells were collected and TCR expression was determined using anti-murine TCRβ by flow cytometry. Briefly, cells were incubated in live/dead Zombie violet dye (BioLegend), Fc blocked with Trustain FcX (BioLegend), washed and stained with a cocktail containing anti-mouse TCRβ (BD horizon) and anti-CD3 antibodies (BioLegend) in FACS staining buffer (Invitrogen).

### Antibody-dependent activation assays

For antibody-mediated functional assays, concentrated supernatant samples of B cell cultures were employed. B cells were engineered on Day 3, as per protocol described above, and cultured for 10 days total and supernatants were harvested and concentrated using AmiconUltra Centrifugal Filter Tubes (50 kDa MWCO) (Millipore) to remove cytokines and large proteins from cell culture. Samples were then quantified for total IgG by ELISA using corresponding recombinant IgG1 antibodies as the standard control. For all experiments, recombinant 6F4 antibody and concentrated supernatant from 6F4 engineered B cells served as the isotype controls.

Flow cytometry-based ADCP assay: THP-1 and PA-1 cells (ATCC) were labelled with 1.25 μM of different CellTrace dyes (Invitrogen) to distinguish effector and target cells. Cells were labelled as described previously (83). In short, cells were labelled in PBS for 4 minutes at 37°C, followed by 4 minutes at room temperature, and then rested for 5 minutes with complete media with serum prior to washing. 5e4 target cells were added to a 96-well round bottom plate along with titrations of recombinant antibodies or concentrated supernatants from engineered B cells culture. Plates were incubated for 10 minutes at room temperature, followed by the addition of 2e4 THP-1 cells for a 1:2.5 E:T ratio. Samples were incubated for 1 hr at 37°C, then washed and fixed with FluoroFix (BioLegend). ADCP% was determined as the frequency of THP-1 cells that took up the PA-1 cell label by flow cytometry; no antibody controls (0 ng/mL) were used to normalize for background phagocytosis by THP-1 cells.

Flow cytometry-based CDC assay: 5e4 PA-1 cells (ATCC) were added to a 96-well round bottom plate along with titrations of recombinant antibodies or concentrated supernatants from engineered B cells culture in media without serum. Plates were incubated for 10 minutes at room temperature, followed by a final concentration of 10% complement-verified human serum (Complement Technology). Samples were incubated for 4 hours at 37°C. Cells were collected with TrypLE (Gibco) and stained with Fixable Live Dead (BioLegend), and fixation with FluoroFix (BioLegend). CDC% was determined as the frequency of dead target cells by flow cytometry; no antibody controls (0 ng/mL) were used to normalize for background cell death.

ADCC reporter assay: the ADCC Reporter Bioassay, V Variant Kit (Promega) was performed per manufacturer’s instructions and incubated for 6 hours using 1.5e4 adherent target cells. Luminescence was determined as relative light units (RLU) using a Varioskan LUX microplate reader (ThermoScientific), which was then used to calculate the fold of induction (FOI). Samples were normalized using background only (media alone) and no antibody control (0 ng/mL).


$$\text{FOI}=\frac{\left(\text{RL}{\text{U}}_{\text{induced}}-\text{RL}{\text{U}}_{\text{background}\;\text{av}}\right)}{\left(\text{RL}{\text{U}}_{\text{no}\;\text{Ab}\;\text{control}\;\text{av}}-\text{RL}{\text{U}}_{\text{background}\;\text{av}}\right)}$$

Flow cytometry-based ADCC assay: frozen NK cells (Stem Cell) were thawed and rested for 24 hours at 1e6 cells/mL in Advanced RPMI (Gibco) supplemented with 10% human AB serum (GeminiBio), 2 mM GlutaMAX (Gibco), 1 mM Sodium Pyruvate (Gibco), 100 IU/mL human IL-2 (PeproTech), and 25 IU/mL human IL-15 (PeproTech) as described by ThermoFisher Scientific. Targets were labelled with 1.25 μM of CellTrace Violet (Invitrogen), as described above, and 2e4 cells/well were added to a 96-well flat bottom TC-treated plate and incubated overnight at 37°C on Day -1 to allow to re-attach. On day of assay, target cells were treated with titrations of recombinant antibodies or concentrated supernatants from engineered B cells culture. Plates were incubated for 10 minutes at room temperature, followed by the addition of 2.75e4 NK cells/well for a 1.4:1 E:T ratio. Samples were incubated for 6 hours at 37°C. Supernatants were transferred to a 96-well round bottom plate, adherent cells collected with TrypLE (Gibco), and pooled with corresponding supernatant samples for staining. Samples were labeled with Fixable Live Dead (BioLegend) and fixed with FluoroFix (BioLegend). ADCC% was determined as the frequency of dead target cells by flow cytometry; no antibody controls (0 ng/mL) were used to normalize for non-specific NK cell killing.

### B cell and dendritic cell antigen presentation assays

B cells from Day 6 of our engineering protocol were washed, resuspended in media, and plated at 2.5e5 cells per well in a 96-well plate. Recombinant E6 protein was added to the wells and B cells were incubated for 20 minutes at 37°C and 5% CO_2_. Freshly thawed donor-matched UTD or E6 TCR T cells were added at a ratio of 1:1. Cells were incubated for 24 hours at 37°C and 5% CO_2_, supernatant was collected, and IFNγ secretion analyzed by MSD (Meso Scale Discovery). For immune complex experiments, day 6 supernatant from B cells that had been engineered with 6F4 BCR or EP-only controls were collected. Supernatants were pre-incubated with increasing concentrations of E6 protein to form immune complexes for 15–30 minutes. To differentiate myeloid DCs, healthy donor PBMCs were cultured following the manufacturer’s protocol (StemCell Technologies). 2.5e5 cells/well of a 96-well plate were incubated with UTD or E6 TCR T cells at a ratio of 1:1 in the presence of immune complexes. Cells were incubated for 24 hours at 37°C and 5% CO_2_, supernatant was collected, and IFNγ secretion analyzed by MSD.

### Caski + mbE6 cell line generation

Caski cells were treated with 8 μg/mL Polybrene and transduced with lentivirus at an MOI of 10, followed by selection with 1 μg/mL Puromycin. E6 was tethered to the membrane by a (G4S)_3_ flexible linker and truncated EGFR. The fusion protein was inserted into a GLV2-EF1a-MCS-PGK-puro vector (Genscript). Successful transduction was determined by flow cytometry using AF647 G4S Linker (E7O2V) antibody (Cell Signaling). Briefly, cells were labeled with Fixable Live Dead and Human TruStain FcX (BioLegend), followed by extracellular staining, and fixation with FluoroFix (BioLegend).

### Membrane-labeled tumor and engineered B cells co-cultures

Suspended PA-1, OV90, OVCAR3, A549, and Caski + mbE6 cells were labelled with a PKH26 red fluorescent cell linker midi kit (Sigma-Aldrich) according to the manufacturer’s protocol. Tumor cells were then plated at 1e5 cells/well in TC-treated 24-well plates to allow them to re-adhere on Day -1. The following day, B cells from Day 6 of engineering protocol were added at 1e5 cells/well for a 1:1 ratio of Targets:B cells. Plates were then incubated for 30 minutes at 37°C. Cells were removed and stained for CD19 expression to distinguish target cells from B cells (clone HIB19, BioLegend) then washed and fixed with FluoroFix (BioLegend). Uptake % was determined as the frequency of B cells that took up the tumor cell label by flow cytometry; samples without tumor cells served as controls to normalize for background.

### Antigen-specific IgG, IgM and IgA ELISPOT

96-well PVDF plates (Millipore) were coated with 100µl capture antibody in PBS (1µg total antibody/well). A different capture antibody was employed for each Ig isotype (IgM: MT11/12, IgA: MT57, IgG MT91/145, MabTech). Plates were incubated overnight at 4°C. Antibody solution was removed the next day and the wells blocked with 1% BSA, Fraction V (Thermo) for 2 hours at 37°C. Blocking solution was then decanted just prior to adding B cells to the wells. B cells from Day 6 of engineering protocol were washed twice in AIMV (Gibco), counted, and resuspended at 1e4/ml in AIMV and 1e5 cells were added. No cell control wells received media alone. Plates were incubated for 18 hrs at 37°C, 5% CO2. The next day, cells were discarded and plates washed vigorously with 0.05% Tween-20 in PBS. The biotinylated peptide for the 6F4 E6 epitope (MHQKRTAMFQDPQERPRKLP) was resuspended in 0.5% BSA fraction V in PBS and plated 100ul/well. Plates were incubated for 2 hours at room temperature, washed with PBS, and 100µl of Streptavidin-HRP (Mabtech) diluted in 0.5% BSA fraction V in PBS was added to each well and incubated for 1 hour at room temperature. Plates were again washed in PBS and 50µl TMB substrate (Mabtech) added per well, following the manufacturer’s instructions. Spot development was then stopped under tap water. Plates were allowed to dry overnight in darkness before being sent to ZellNet Consulting for analysis.

### Tumor cell labeling

CLDN6-expressing tumors were stained with Fixable Live Dead and Human TruStain FcX (BioLegend), followed by human CLDN6 AF647-conjugated antibody (R&D Systems, clone 342927) and fixation with FluoroFix (BioLegend).

### Tumor and T cell activation assays

1e5 tumor cells were plated/well in TC-treated 24-well plates to allow them to re-adhere on Day -1. The following day, 1e5 B cells from Day 6 of engineering protocol were added and incubated for 30 minutes, prior to the addition of 1e5 freshly thawed UTD or E6 TCR T cells for a 1:1:1 ratio of Targets:B cells:T cells. For soluble controls, 1e5 B cells were added to a 96-well plate and incubated with 0 or 100 nM E6 protein (R&D Systems) or 1 μg/well E6_1–15_ peptide (Genscript) for 20 minutes, followed by the addition of 1e5 UTD or E6 TCR T cells for a 1:1 ratio of B Cells:T cells. All plates were incubated for 20 hours at 37°C, followed by 4 hours with 1X Brefeldin A (BioLegend). Cells were removed and labeled with Fixable Live Dead and Human TruStain FcX (BioLegend), followed by extracellular staining for CD3, CD4, CD8, and CD19 (BioLegend). Intracellular IFNγ (BioLegend) staining was then performed using Cyto-Fast Fix/Perm Buffer Set and Human TruStain FcX (BioLegend).

## Data Availability

The raw data supporting the conclusions of this article will be made available by the authors, without undue reservation.
