# Supplementary material for: Engineered human B cells targeting tumor-associated antigens exhibit antigen presentation and antibody-mediated functions
Source: Front Immunol. 2025 Jul 30;16:1621222. doi: 10.3389/fimmu.2025.1621222 (PMC12343648; doi:10.3389/fimmu.2025.1621222)
Supplement: Supplementary file 1 [file DataSheet1.pdf]

Title

Engineered Human B Cells Targeting Tumor-Associated Antigens Exhibit Antigen Presentation and Antibody-Mediated Functions

Authors

Alexander Boucher[1,†], Courtney Anderson[2,†], Rochelle Hinman[2], Molly Kindschuh[2], Jeremy Fung[2], Tiansu Wang[1], Isabella Klooster[1], Elise Kim[1], Caroline Roth[3], Michael Vander Oever[3], Bakhmala Khan[1], Natalie Zelikson[4], Yaron Vagima[5], Huseyin Saribasak[1], Lisa Santry[1], Leah Natasha Klapper[6], Shmuel Hess[6], Jill Mooney[1], Débora Rosa Bublik[6], Haley Laken[2], Adi Barzel[7], Philip Borden[3], Cherylene Plewa[1], Ana Maria Chadbourne[1,†], Devin Bridgen[2,†], Alessio D. Nahmad[6,8,†,*]

1: ElevateBio, Waltham, MA, United States of America
2: Tabby Therapeutics, Watertown, MA, United States of America
3: Life Edit Therapeutics, Durham, NC, United States of America
4: Faculty of Medical & Health Sciences, Tel Aviv University, Tel Aviv, Israel
5: Department of Biotechnology, Israel Institute for Biological Research, Ness Ziona, Israel
6: Tabby Therapeutics, Ness Ziona, Israel
7: Faculty of Life Sciences, Tel Aviv University, Israel
8: The Samueli Integrative Cancer Pioneering Institute, Petah Tikva, Israel

Alexander Boucher[1,†], Courtney Anderson[2,†] : *These authors contributed equally to this work and share first authorship*

Ana Maria Chadbourne[1,†], Devin Bridgen[2,†], Alessio D. Nahmad[6,8,†,*]: *These authors share last authorship*

Alessio D. Nahmad[6,8,†,*]: *corresponding author alessionahmad@gmail.com*

Supplementary figures and legends:

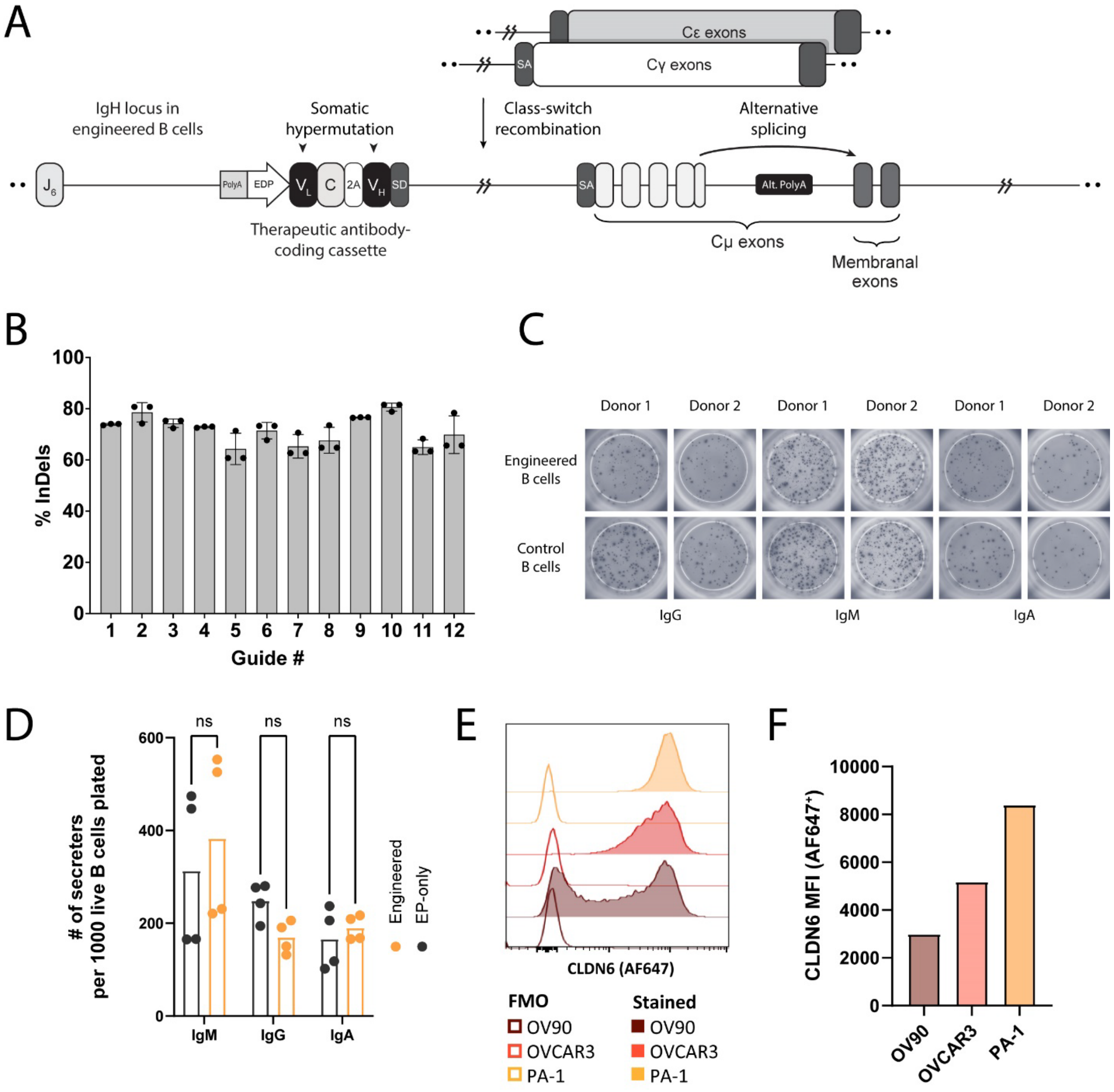

Supplementary Figure 1:

A. Schema depicting the engineered IgH target locus in B cells. The cassette is integrated between the last J segment and the endogenous constant exons. The cassette encodes a polyadenylation site (PolyA) to ablate endogenous expression of the heavy chain and a promoter (EDP) drives transcription upon on-target integration. The light chain of the therapeutic antibody is coded by both the variable light (V_L), light chain constant (C) and the variable heavy (V_H). A splice donor (SD) in the cassette reacts with the splice acceptor (SA) of the endogenous constant segments to form a full heavy chain. A 2A peptide separates the

light chain from the heavy chain during translation. Because the cassette is integrated upstream of the class-switch recombination sites, it may be expressed with multiple isotypes. Concordantly, an alternative polyadenylation site (Alt. PolyA) is active in antibody secreting cells, ablating transcription of the membranal exons of the constant domain. Thus, the integrated cassette may express the therapeutic antibody either as a BCR or as a soluble antibody, depending on the cell differentiation status. **B.** Editing rates of lead guides identified by screening using Nuclease A. Data are from three independent experiments. Error bars indicate SD. **C.** ELISPOT for antigen non-specific, total IgM, IgG and IgA secreted by B cells engineered to express the anti-E6 6F4 antibody compared to EP-only control B cells. **D.** Quantification of A. ns=pv>0.05 for two-way ANOVA with Šídák's multiple comparisons test. **E.** CLDN6 antigen expression on OV90 (brown), OVCAR3 (red) and PA-1 (orange) cell lines, as determined by flow cytometry. Fluorescence minus one (FMO, outlined histograms) is compared to stained cells (filled histograms). **F.** Quantification of CLDN6 antigen mean fluorescence intensity (MFI) using of CLDN6^{+} population.

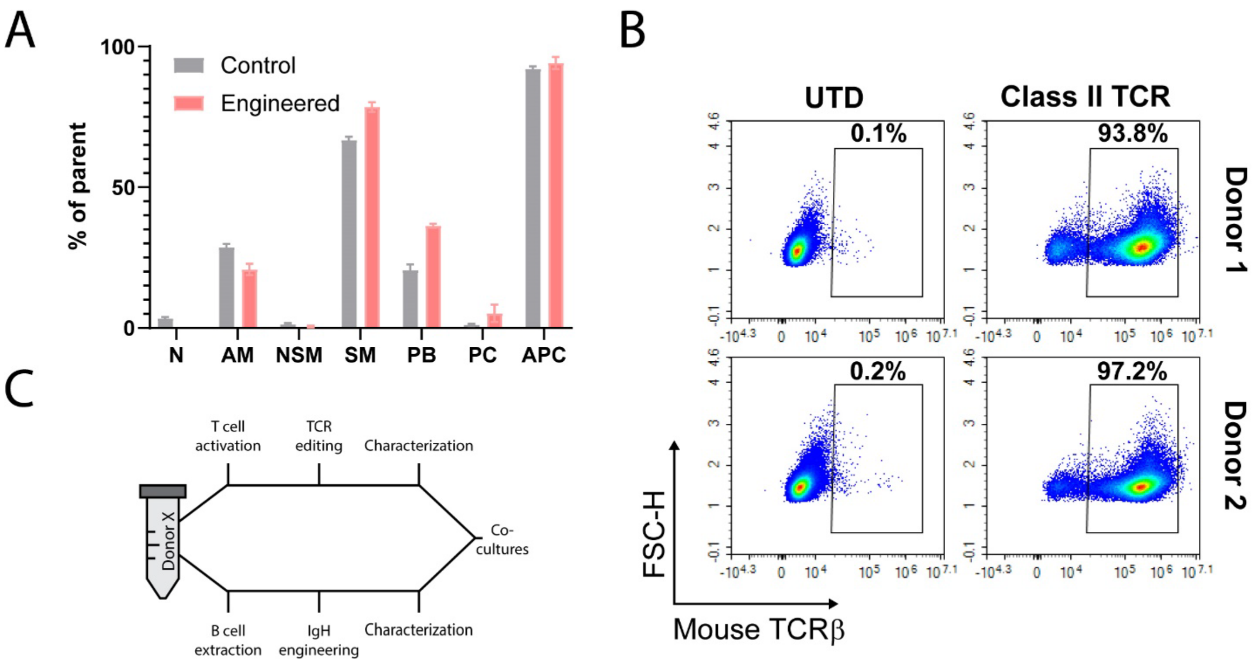

Supplementary Figure 2:

A. B cell subsets for C1P5-engineered or non-electroporated control B cells were determined by flow cytometry. Subsets were as follows: Naïve (N) IgD^{+}CD27^{-}, Atypical Memory (AM) IgD^{-}CD27^{-}, Non-Switched Memory (NSM) IgD^{+}CD27^{+}, Switched Memory (SM) IgD^{-}CD27^{+}, Plasmablast (PB) CD27^{+}CD38^{+}, Plasma Cell (PC) CD38^{+}CD138^{+}, Antigen-Presenting Cell (APC) CD86^{+}CD21^{-}. **B.** Representative TCR expression of E6 TCR T cells from two donors. The T cells were transduced with a lentiviral vector coding for E6 Class II-restricted TCR expressing murine constant segments to avoid mispairing and enable detection. Cells are compared to untransduced (UTD) cells. Transduction is determined by flow cytometry using an antibody specific for murine β constant region of the TCR. **C.** Schema depicting antigen presentation assays. B cells or T cells

from the same donors are extracted, engineered and co-cultured in the presence of the tumor-associated antigen.

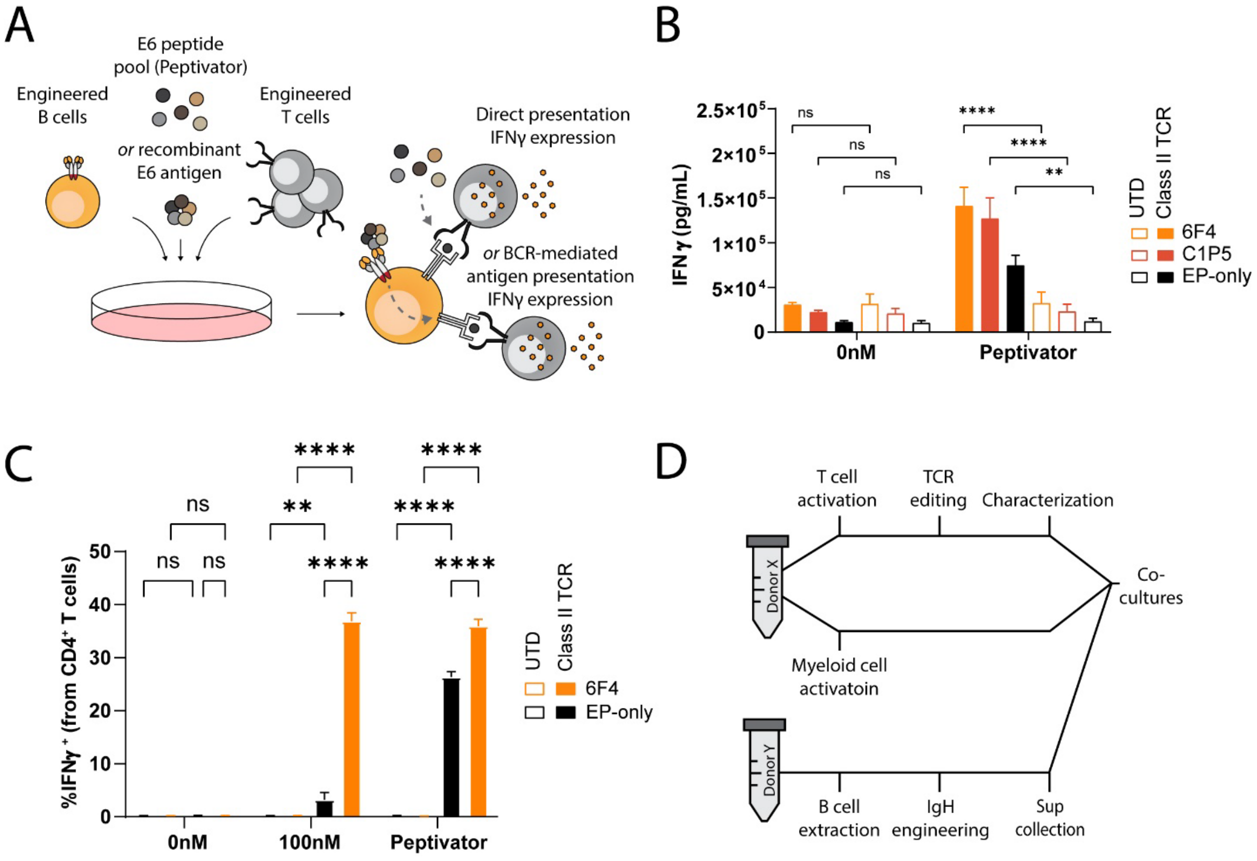

Supplementary Figure 3:

A. Schema depicting engineered B cell antigen presentation assay. B cells, either non-engineered or engineered to express anti-E6 antibodies, are loaded with recombinant E6 antigen at multiple concentrations or E6 peptide pools and then incubated with T cells, either engineered or non-engineered to express an anti-E6 TCR. The concentration of IFNγ in the supernatants is then analyzed by ELISA. Alternatively, the frequency of T cells expressing intracellular IFNγ is then analyzed by flow cytometry. **B.** ELISA for IFNγ as in A. and as in Figure 4B. In this case, the B cells were either incubated without antigen or with an E6 peptide pool (Peptivator), instead of the full antigen. Data representative of 2 experiments with 2 donors. Bars and error bars represent mean and SD. ns=pv>0.05, **=pv<0.01, ****=pv<0.0001 for two-way ANOVA with Šídák's multiple comparisons test. **C.** Intracellular flow cytometry for IFNγ in CD4+ T cells as in A. B cells were either unloaded (0nM E6), loaded with 100nM E6 or loaded with E6 peptide pools (Peptivator), instead of the full antigen. Data representative of 2 independent experiments. For B-C, bars and error bars represent mean and SD. ns=pv>0.05, **=pv<0.01, ****=pv<0.0001 for two-way ANOVA with Šídák's multiple comparisons test. **D.** Schema depicting immune complex presentation assays. Dendritic cells are differentiated from myeloid cells and T cells from the same donors are extracted,

engineered and co-cultured in the presence of immune complexes. The immune complexes are formed by soluble antigen with antibodies from concentrated antibodies from the supernatants of engineered B cells.
