# Supplementary figures and images for: Engineered human B cells targeting tumor-associated antigens exhibit antigen presentation and antibody-mediated functions
Source: Front Immunol. 2025 Jul 30;16:1621222. doi: 10.3389/fimmu.2025.1621222 (PMC12343648; doi:10.3389/fimmu.2025.1621222)

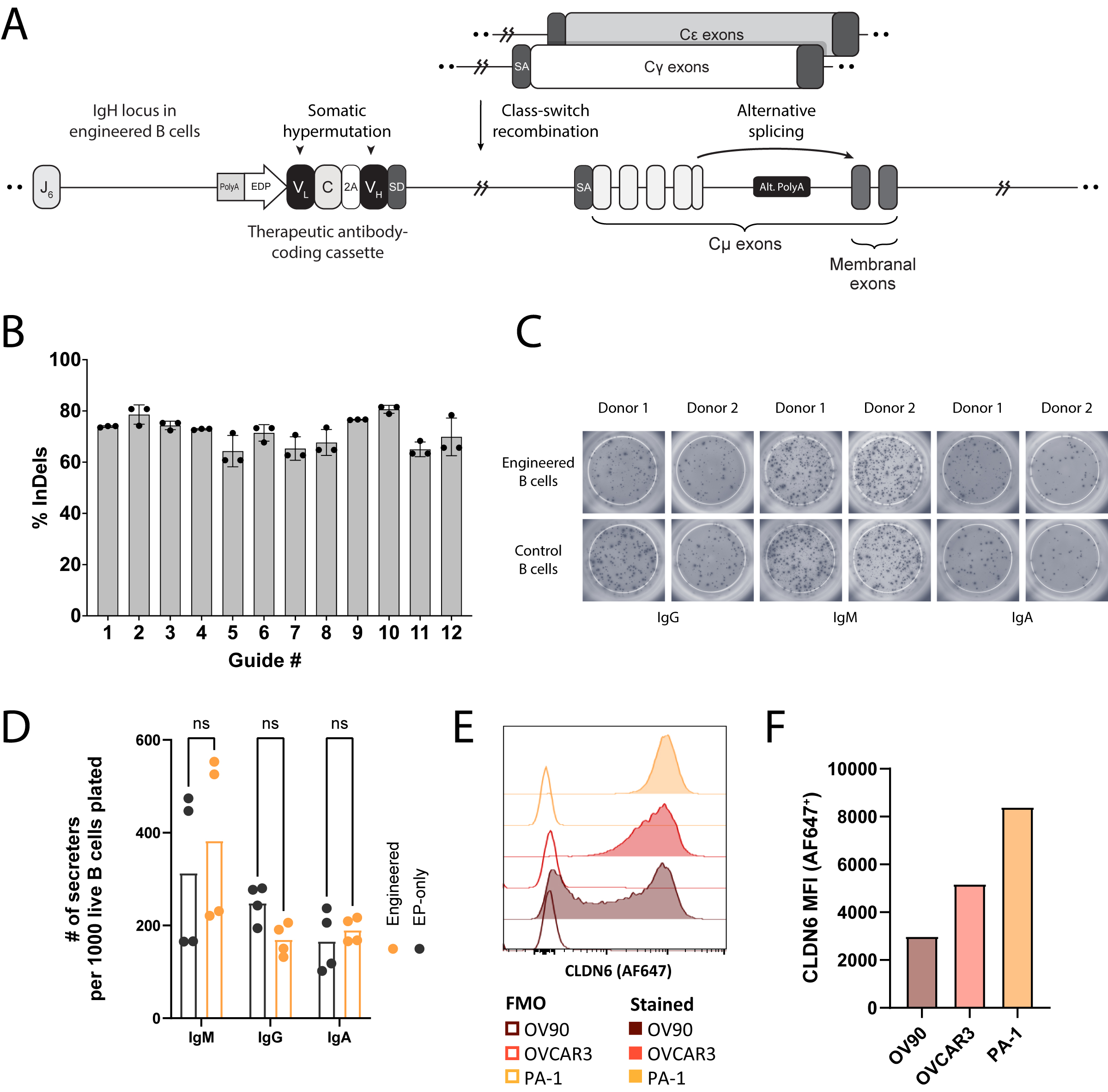

Supplement: Supplementary file 3 [file Image1.jpeg]

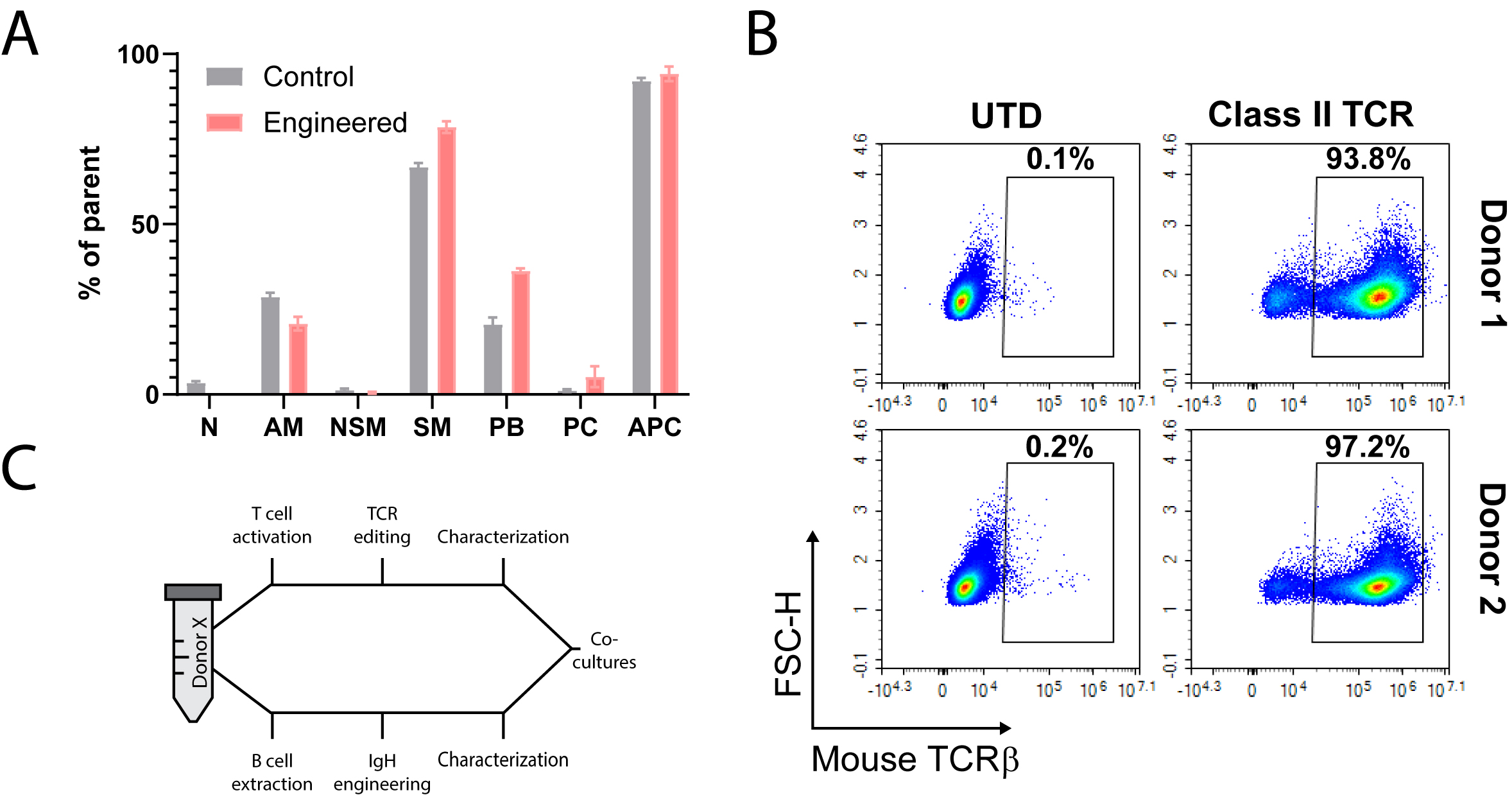

Supplement: Supplementary file 4 [file Image2.jpeg]

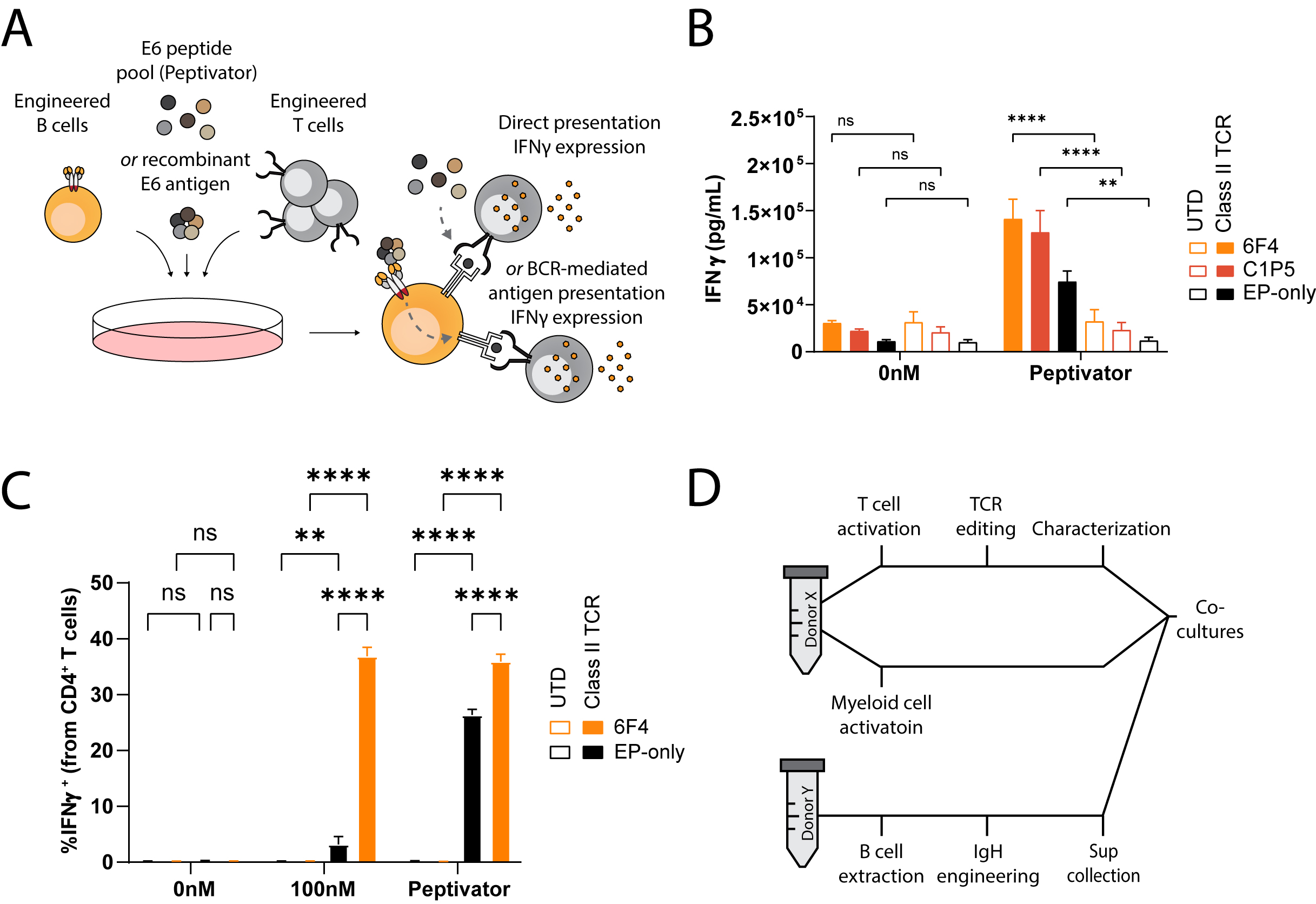

Supplement: Supplementary file 5 [file Image3.jpeg]
